# United States Department of Agriculture

**Published:** 1897-10

**Authors:** A. J. Wedderburn, James Wilson

**Affiliations:** Special Agent; Washington, D. C.; Secretary; Washington, D. C.


### UNITED STATES DEPARTMENT OF AGRICULTURE.

### DIVISION OF CHEMISTRY.

WASHINGTON, D. C., September 17th, 1897.

DEAR SIR:

By direction of Congress, the Department of Agriculture is investigating the character and extent of the adulteration of foods and drugs. It is generally believed that adulteration, sophistication, imitation, and misbranding of foods, drugs, and liquors exist to a very great extent. Many of the States have enacted laws to prevent such practices, and it is very desirable to know how these laws have been enforced, and with what results.

As the general public is largely interested in this matter, as it affects health, morals, and legitimate trade, it is thought proper to ask the co-operation of the press in securing accurate information on the subject. The publication of a simple request for information on this subject, to be furnished the paper asking it, or sent direct to the Chemical Division of the Department of Agriculture, will in all probability secure a large amount of valuable *data* which will materially assist in properly carrying out the work. As no matter can be of more importance to the people of the United States than that of the extent and character of the adulteration of foods and drugs sold them, I take the liberty of asking your co-operation in the work as herein indicated. Please state that the Department simply desires a concise statement of facts, which can be fully substantiated if necessary, and not theories.

Respectfully,

A. J. WEDDERBURN,

*Special Agent.*

Approved:

JAMES WILSON,

*Secretary.*

